# Bacterial and Archaeal α-Amylases: Diversity and Amelioration of the Desirable Characteristics for Industrial Applications

**DOI:** 10.3389/fmicb.2016.01129

**Published:** 2016-07-28

**Authors:** Deepika Mehta, Tulasi Satyanarayana

**Affiliations:** Department of Microbiology, University of DelhiNew Delhi, India

**Keywords:** archaea, bacteria, dextrinizing α-amylases, saccharogenic α-amylases, protein engineering, native and recombinant α-amylases

## Abstract

Industrial enzyme market has been projected to reach US$ 6.2 billion by 2020. Major reasons for continuous rise in the global sales of microbial enzymes are because of increase in the demand for consumer goods and biofuels. Among major industrial enzymes that find applications in baking, alcohol, detergent, and textile industries are α-amylases. These are produced by a variety of microbes, which randomly cleave α-1,4-glycosidic linkages in starch leading to the formation of limit dextrins. α-Amylases from different microbial sources vary in their properties, thus, suit specific applications. This review focuses on the native and recombinant α-amylases from bacteria and archaea, their production and the advancements in the molecular biology, protein engineering and structural studies, which aid in ameliorating their properties to suit the targeted industrial applications.

## Introduction

Starch is a glucose polymer, which is synthesized by a wide array of plant species. Starch granules contain of two types of α-glucans, amylose and amylopectin, overall representing 98–99% of its total dry weight. Amylose is a linear water insoluble polymer of glucose joined by α-1, 4 glycosidic bonds (99%), while amylopectin is branched water soluble polysaccharide with short α-1, 4 linked linear chains of 10–60 glucose units and α-1, 6 linked side chains with 15–45 glucose units that form the volume of starch molecule (Buleon et al., 1998; Tester et al., 2004). The ratio of the two polysaccharides depends on the botanical origin of the starch, but the representative levels of amylose to amylopectin are 25–28 and 72–75%, respectively.

Based on the mode of action, starch hydrolyzing enzymes may be endo-acting or exo-acting. α-Amylases (EC 3.2.1.1), categorized in GH-13 family of glyosyl hydrolases, are the extracellular endo-acting enzymes that hydrolyse α-1,4 glycosidic linkages of starch randomly, while bypassing the branch points and liberating α-limit dextrins as products (Antranikian, 1992; Gupta et al., 2003; Sivaramakrishnan et al., 2006). Amylolytic enzymes have been elegantly reviewed by several workers earlier (Vihinen and Mantasala, 1989; Henrissat, 1991; Antranikian, 1992; Svensson, 1994; Davies and Henrissat, 1995; Guzman-Maldonadao and Paredes-Lopez, 1995; Nigam and Singh, 1995; Crabb and Mitchinson, 1997; Janecek, 1997; Janecek et al., 1997, 1999; Sunna et al., 1997; Bisgaad-Frantzen et al., 1999; Pandey et al., 2000; Vieille and Zeikus, 2001; van der Maarel et al., 2002; Gupta et al., 2003; Rubin-Pitel and Zhao, 2006; Sivaramakrishnan et al., 2006). Another category belongs to carbohydrases that hydrolyze cyclodextrins. They are called maltogenic amylases (EC 3.2.1.133), which are catalytically versatile and can hydrolyze α-(1,4)- as well as α-(1,6)- linkages of the substrate molecule and transglycosylate the hydrolytic products. They possess an additional 130 residues at the N-terminus that are absent in the typical α-amylases. They can also hydrolyze acarbose, a potent inhibitor of α-amylases, to produce glucose and acarviosine-glucose [pseudotrisaccharide (PTS); (Li et al., 2011)]. β-Amylase (E.C.3.2.1.2) is an exoenzyme that hydrolyzes every alternate α-1,4 linkage from the non-reducing end, causing inversion of the anomeric configuration of the liberated maltose to its β-form, and hence, they are called β-amylases. This enzyme bypasses α–1,6-linkages of branched substrates producing maltose and high molecular weight β-limit dextrins (Vihinen and Mantasala, 1989). In comparison, glucoamylase (E.C.3.2.1.3), also known as amyloglucosidase or γ–amylase, slowly acts on α–1,4 linkages of α-glucans from the non-reducing ends and also α–1,6 linkages. It preferentially degrades polysaccharides with a high molecular weight. These enzymes hydrolyze starch to yield glucose in theoretically 100% yield (Kumar and Satyanarayana, 2004). CGTase (E.C.2.4.1.19) produces a series of α, β, γ cyclodextrins from starch, amylose, and other polysaccharides. In addition, CGTases also catalyze coupling reaction by which the rings are opened and transferred to co-substrates like glucose, maltose, or sucrose. The enzyme also catalyzes disproportionation reaction in which one or more glycosyl units are transferred between linear oligosaccharides. A hydrolysis reaction is also catalyzed by the enzyme that produces dextrins. Another starch hydrolyzing enzyme α–glucosidase (E.C.3.2.1.20) acts on α–1,4-and/or α–1,6-linkages of oligosaccharides from non-reducing end, which are formed by the action of other amylolytic enzymes, liberating α–D glucose units. Most enzymes show high affinity toward maltose and so they are called as maltases (Vihinen and Mantasala, 1989; Gupta et al., 2003; Sivaramakrishnan et al., 2006).

According to international starch market index, the total utilization of starch in the world in 2008 was 66 million tons. This increased to 75 million tons by 2012, which suggests an annual growth rate of 2–3%. Fructose syrups represent 72% of starch output, while ethanol alone 40% (http://starch.dk/isi/market/index.asp). The industrial enzyme market was valued at USD 4.2 billion in 2014 and is estimated to reach USD 6.2 billion by 2020. The food and beverage segment of industrial enzymes market is estimated to reach US$ 2.0 billion by 2020 (http://www.marketsandmarkets.com/PressReleases/industrial-enzymes.asp). Among industrial enzymes, starch hydrolyzing enzymes find applications in the production of ethanol, HFCS (high fructose corn syrup), and detergent and baking industries. Alcohol and starch enzyme market is estimated to be worth $2.24 billion by 2018 (http://www.marketsandmarkets.com/PressReleases/alcohol-starch-sugar-enzyme.asp).

## Diversity of α-amylases

α-Amylases are divided into two categories according to the degree of hydrolysis. Saccharifying α-amylases produce free sugars and reduce the viscosity less rapidly in comparison with the amount of reducing sugars released. While liquefying α-amylases, on the other hand, break down the starch polymer, but do not produce free sugars and cause more rapid reduction in viscosity of starch pastes. α-Amylases have bacterial, archaeal, and eukaryotic origin. The bacterial α-amylases are quite distant from the eukaryotic enzymes evolutionarily. Among all α-amylases, the bacterial enzymes are the most diverse (Pandey et al., 2000). The temperature-activity optima for bacterial α-amylases range from ~25°C (Feller et al., 1992; AHA) to around 100°C (Rao and Satyanarayana, 2007b; *Geobacillus thermoleovorans* α-amylase). The pH optima of bacterial α-amylases vary from 1 to 11.5 (Vihinen and Mantasala, 1989; Pandey et al., 2000). α-Amylase from *Bacillus* sp. and *Alicyclobacillus acidocaldarius* showed an acidic pH optima of 1 and 3, respectively (Uchino, 1982; Schwermann et al., 1994). In contrast, alkaline amylase with optima of pH 9–10.5 had been reported from many alkaliphilic *Bacillus* spp. (Saito, 1973; Krishnan and Chandra, 1983; Lee et al., 1994; Shinke et al., 1996). An extremely alkaline α-amylase with pH optima of 11.5 was reported from *Bacillus* sp. GM8901 (Kim et al., 1995). Archaeal α-amylases, in general, are thermostable and acidic (pH 5–6) in nature. For example the highest temperature optimum has been reported as 100 and 130°C from archaea, *Pyrococcus furiosus* and *P. woesei*, respectively (Koch et al., 1991; Laderman et al., 1993). Molecular weight of α-amylases vary from about 10 to 210 kDa, 10 kDa enzyme from *Bacillus caldolyticus* (Grootegoed et al., 1973) and the highest molecular weight (210 kDa) from *Chloroflexus aurantiacus* has been reported (Ratanakhanokchai et al., 1992). Table 1 presents temperature and pH optima and molecular weights of α-amylases from bacteria and archaea.

**Table 1:** **Characteristics of bacterial and archaeal α-amylases**.

**Source**	**Molecular weight**	**pH optimum**	**Temperature optimum (°C)**	**K_m_, V_max_**	**References**
*Acyclobacillus acidocaldarius*	160	3.0	75	–	Matzke et al., 1997
*Alicyclobacillus* sp. A4	64	4.2	75	–	Bai et al., 2012
*Amphibacillus* sp. NM-RA2	–	8	54	–	Mesbah and Wiegel, 2014
*Bacillus acidicola*	66	4	60	–	Sharma and Satyanarayana, 2010
*B*. *amyloliquefaciens*	52	6.0	55	1.92 mg ml^−1^, 351 Uml^−1^	Demirkan et al., 2005
*B*. *amyloliquifaciens*	43	7	70	–	Kikani and Singh, 2011
*B. circulans*	48	4.9	48	11.66 mg ml^−1^, 68.97U	Dey et al., 2002
*B*. *halodurans*	90, 85, 70, 65, 58	10.5–11	60–65	–	Murakami et al., 2008
*B*. *licheniformis*	31	6.5	90	–	Bozic et al., 2011
*B. licheniformis*	58	4–9	90	–	Hmidet et al., 2008
*B. stearothermophilus*	59	5.6	80–82	–	Ali et al., 2001
*B. stearothermophilus*	56	5.6	80	–	Khemakhem et al., 2009
*B*. *subtilis* AS-S01a	21	9	55	1.9 mg ml^−1^, 198.2 U ml^−1^	Roy et al., 2012
*B. subtilis* KIBGE	56	7.5	50	2.68 mg ml^−1^, 1773 U ml^−1^	Bano et al., 2011
*Bacillus* sp.	–	8	110	–	Pancha et al., 2010
*Bacillus* sp. A3-15	86	8.5	60	–	Burhan, 2008
*Bacillus* sp. ANT-6	94.5	10.4	80	–	Burhan et al., 2003
*Bacillus* sp. KR8104	59	4.0–6.0	75–80	–	Sajedi et al., 2005
*Bacillus* sp. TS-23	65	9	60	–	Lo et al., 2001
*Bacillus* sp. WPD616	59	6	50	–	Liu et al., 2006
*Bacillus* sp. YX1	56	5.0	40–50	–	Liu and Xu, 2008
*Bacillus* sp.	53	4.5	70	–	Asoodeh et al., 2010
*Bacillus subtilis* JS-2004	–	7	50	–	Asgher et al., 2007
*Chromohalobacter* sp.	62, 72	9	65	–	Prakash et al., 2009
*Chryseobacterium taeanense*	47	9	50	–	Wang et al., 2011
*Desulfurococcus mucosus*	–	5.5	100	–	Canganella et al., 1994
*Geobacillus caldoxylosilyticus* TK4	70	7	50	–	Kolcuoglu et al., 2010
*G. thermodenitrificans*	58	5.5	80	3.05 mg ml^−1^, 7.35 Uml^−1^	Ezeji and Bahl, 2006
*G*. *thermoleovorans*	26	7	100	–	Rao and Satyanarayana, 2007a
*Geobacillus* sp. IIPTN	97	6.5	60	36 mg ml^−1^, 222 Uml^−1^	Dheeran et al., 2010
*Geobacillus* sp. LH8	52	5–7	80	–	Khajeh et al., 2009
*Geobacillus* sp. LH8	52	5–7	80	3 mg ml^−1^, 6.5 μ mol min^−1^	Mollania et al., 2010
*Halomonas meridiana*	–	7.0	37	–	Coronado et al., 2000a
*Lactobacillus manihotivorans*	135	5.5	5	–	Aguilar et al., 2000
*Marinobacter* sp. EMB8	72	6–11	80	4.6 mg ml^−1^, 1.3 mg min^−1^ml^−1^	Kumar and Khare, 2012
*Nesterenkonia* sp. strain F	100	7.5	45	–	Shafiei et al., 2010
*Pyrococcus furiosus*	48	5.6	115	–	Savchenko et al., 2002
*P. woesei*	45	5.5	95	–	Frillingos et al., 2000
*Saccharopolyspora* sp. A9	66	11	55	–	Chakraborty et al., 2011
*Staphylothermus marinus*	82.5	5	100	–	Li et al., 2010
*Sulfolobus solfataricus*	120	3	80	–	Haseltine et al., 1996
*Thermococcus* sp. CL1	–	6	98	–	Jeon et al., 2014
*T. aggregans*	–	5.5	100	–	Canganella et al., 1994
*T. celer*	–	5.5	90	–	Canganella et al., 1994
*T. guaymagensis*	–	6.5	100	–	Canganella et al., 1994
*T. hydrothermalis*	–	5.0–5.5	75–85	–	Legin et al., 1998
*T. hydrothermalis*	53.5	5.5	85	–	Horvathova et al., 2006
*T. profundus*	43	5.5	80	–	Kwak et al., 1998
*Thermotoga maritima*	50	7	70	–	Lim et al., 2003

Increased demands for α-amylases with desired properties for industrial applications also encouraged exploration from metagenomes from different habitats. The screening of clones from a soil metagenomic library constructed in pUC 19 vector led to find a putative amylase gene (*amyM*), which was over expressed and purified. This enzyme was optimally active at 42°C and pH 9.0 with transglycosylation activity and requirement for Ca^2+^ for its stability (Yun et al., 2004). In another attempt, a gene (pAMY) of 909 bp encoding an α-amylase was found when a soil-derived metagenomic library was screened. Phylogenetic analysis revealed that pAMY was closely related to amylases from uncultured bacteria. The enzyme with a molecular mass of 38 kDa exhibited optimum activity at 40°C and neutral pH (Sharma et al., 2010). A function-based screening of an acid mine drainage (AMD) derived metagenomic library led to the discovery two endo-acting amylases which shared no sequence similarity with any known amylase. They didn't have known amylolytic domains or could be assigned to any GH-family (Delavat et al., 2012). A thermostable α-amylase gene was isolated from a metagenomic library constructed from metagenome constructed from a pilot-plant biogas reactor. When the gene (1461 bp) was cloned in *Escherichia coli*, a very high titre was attained. Further characterization revealed that it has optimal activity at 80°C and highly salt tolerant [25% (w/v) NaCl]. This novel enzyme Amy13A showed 75% sequence identity to α-amylases from *Petrotoga mobilis* and *Halothermothrix orenii* (Jabbour et al., 2013). Vester et al. (2015) identified a cold-active α-amylase by a functional metagenomics approach. Sequence analysis revealed that the enzyme was similar to α-amylase from the class Clostridia. Its temperature and pH optima were 10–15°C and pH 8–9. α-Amylases have also been reported from metagenomic libraries from pygmy loris (*Nycticebus pygmaeus*) and cow dung (Xu et al., 2014; Sharma et al., 2015).

## Production and characterization of α-amylases

### Production of bacterial and archaeal α-amylases

The production of α-amylases has been studied extensively in submerged and/or solid state fermentations. In general, extracellular α-amylase production is growth associated (Murthy et al., 2009; Asoodeh et al., 2010; Abou Dobara et al., 2011). Among the physical parameters, the temperature and pH of the medium play an important role in α-amylase production. The influence of temperature on α-amylase production is related to the growth of the organism. Amylase production in bacteria has been studied in a wide range of temperatures. α-Amylase production has been reported from thermophilic and hyperthermophilic bacteria and archaea like *Pyrococcus, Thermococcus*, and *Sulfolobus* species (Leuschner and Antranikian, 1995; Sunna et al., 1997), *G. thermoleovorans* (Rao and Satyanarayana, 2007a), *B. acidocaldarius* (Buonocore et al., 1976), and *Alicyclobacillus* sp. A4 (Bai et al., 2012), from mesophiles *B. amyloliquifaciens* (Gangadharan et al., 2008), *Halomonas meridiana* (Coronado et al., 2000a) *B. subtilis* (Ravindar and Elangovan, 2013), *Lactobacillus plantarum* (Kanpiengjai et al., 2015) as well as from psychrotolerants and psychrophiles *Microbacterium foliorum* GA2 (Roohi and Kuddus, 2014) and *Aeromonas veronii* NS07 (Samie et al., 2012). Among different carbon sources used, starch, fructose, glucose, and rice flour, are known to support high enzyme production (Ezeji et al., 2005; Prakash et al., 2009). Carbon sources like glucose and maltose have been used for the production of α-amylase, but the use of starch remains ubiquitous (Mamo and Gessesse, 1999; Sajedi et al., 2005; Liu and Xu, 2008; Sharma and Satyanarayana, 2010). Industrially important enzymes have traditionally been produced in submerged fermentation, but these enzymes are also produced by solid state fermentation. Sodhi et al. (2005) and Hashemi et al. (2010) explained the use of wheat bran for the production of α-amylase. Agro-residues were used for cold-active α-amylase production from *M. foliorum* (Roohi and Kuddus, 2014). In general α-amylase production is inducible in nature (Aiyer, 2004; Ryan et al., 2006; Asoodeh et al., 2010; Abou Dobara et al., 2011), but in few cases α-amylase production is also constitutive (Rao and Satyanarayana, 2007a). Like most other inducible enzymes, α-amylase production is subjected to catabolite repression by maltose and glucose, starch hydrolytic products (Bhella and Altosaar, 1988; Morkeberg et al., 1995) with the exception of some *Bacillus* strains (Kalishwaralal et al., 2010). Nitrogen source is a major factor that affects α-amylase production. Many investigators had confirmed that organic nitrogen sources support maximum α-amylase yields (Saxena et al., 2007; Aqeel and Umar, 2010; Abou Dobara et al., 2011).

The combination of low molecular weight dextran with Tween-80 increased 27-fold higher α-amylase production (Arnesen et al., 1998). Various metal ions like Ca^2+^, Fe^2+^, Mg^2+^, and K^+^ are added to the α-amylase production medium (Sajedi et al., 2005; Liu and Xu, 2008). Phosphate is a vital requirement for microbes as it regulates the synthesis of primary and secondary metabolites. Lower and higher levels of phosphate in the medium significantly affect the growth and enzyme production (Hillier et al., 1997; Sharma and Satyanarayana, 2010).

The production of amylases by microbes is affected considerably by physical and chemical parameters of the medium (Gigras et al., 2002; Rao and Satyanarayana, 2007a; Roohi and Kuddus, 2014; Sen et al., 2014). Traditionally “one-variable-at-a-time” approach has been used (Gokhale et al., 1991; Pham et al., 1998), but it is time consuming and does not permit understanding interactions among the process parameters. The statistical Plackett and Burman design, on the other hand, allows screening of critical culture variables (Sharma and Satyanarayana, 2006; Rao and Satyanarayana, 2007a; Kumar and Satyanarayana, 2008; Roohi and Kuddus, 2014), and response surface methodology (RSM) provides information about the optimum levels of each variable, interactions among them and their effects on the product yield (Gu et al., 2005; Rao and Satyanarayana, 2007a). The statistical approaches have been proved to be useful in optimizing medium components and cultural variables for maximizing amylase titres from many organisms like *B. acidicola* (Sharma and Satyanarayana, 2011), *Bacillus* sp. KR 8104 (Hashemi et al., 2010), and *G. thermoleovorans* (Rao and Satyanarayana, 2007a), *Alcaligenes faecalis* (Sen et al., 2014), and *M. foliorum* (Roohi and Kuddus, 2014).

### Characterization of bacterial and archaeal α-amylases

Once produced and purified, an enzyme is biochemically characterized. α-Amylases show a wide range of substrate degradation. They degrade amylose, amylopectin, cyclodextrins, glycogen, and dextrins, but they possess highest specificity toward starch (Antranikian, 1992). Various metal ions influence activity of the enzymes (Vihinen and Mantasala, 1989). α-Amylase is a metal activated enzyme and has high affinity for Ca^2+^. In, general, Ca^2+^ enhances the activity and thermal stability of most of the α-amylases (Khajeh et al., 2001). The number of bound Ca^2+^ varies from 1 to 10. Dialysis against EDTA can remove the bound Ca^2+^. The Ca^2+^-free amylase can be reactivated by adding Ca^2+^. Although most of the α-amylases are Ca^2+^-dependent, there are reports of Ca^2+^-independent α-amylases too (Sajedi et al., 2005; Rao and Satyanarayana, 2007b; Hmidet et al., 2008; Asoodeh et al., 2010; Sharma and Satyanarayana, 2010). Besides Ca^2+^-independent α-amylases, there are also a few α-amylases, which are inhibited by Ca^2+^ ions (Babu and Satyanarayana, 1993; Tanaka and Hoshino, 2003; Mehta and Satyanarayana, 2013a). The metal ions, which inhibit α-amylase activity, include Hg^2+^ ions (Mamo and Gessesse, 1999; Asoodeh et al., 2010). Inhibition of α-amylase by Hg^2+^ ions indicates the presence of carboxyl groups in enzyme molecule (Dey et al., 2002). Furthermore, Hg^2+^ is also known to oxidize indole ring and to interact with aromatic ring present in tryptophan (Zhang et al., 2007; Liu et al., 2010). Various inhibitors such as dithiothreitol, β-mercaptoethanol, N-bromosuccinimide (NBS), p-hydroxy mercuribenzoic acid, iodoacetate, PMSF (phenylmethylsuphonyl fluoride), Woodward's reagent K, EDTA, and EGTA have been shown to inhibit α-amylases (Hamilton et al., 1999). Inhibition of enzyme activity by NBS demonstrates the role of tryptophan in maintaining the conformational stability of the enzyme (Rao and Satyanarayana, 2007b). Dithiothreitol and β-mercaptoethanol are the reducing agents, the effects of which suggest the role of—SH groups in the catalytic activity of enzyme. DTT may stimulate or inhibit the α-amylase (Ballschmiter et al., 2006; Rao and Satyanarayana, 2007b). In maltogenic α-amylase of *Bacillus* sp. WPD616, DTT had no effect on the α-amylase activity indicating that—SH groups have no role to play in the catalytic activity or these enzymes have no free and accessible—SH groups (Liu et al., 2006). The inhibition of α-amylase by PMSF indicates the role of seryl hydroxyl group in enzyme activity. Woodward's reagent K (WRK) has a role in the chemical modification of aspartic and glutamic acid residues (Paoli et al., 1997). The inactivation of amylase by WRK also indicates the involvement of acidic amino acids in the active site of the enzyme (Chauthaiwale and Rao, 1994; Komissarov et al., 1995; Sharma and Satyanarayana, 2010).

## Gene cloning and over expression of recombinant α-amylases

Several attempts have been made on cloning α-amylase encoding genes from several bacteria and archaea in heterologous hosts such as *E. coli*. A thermostable α-amylase gene of 1203 bp encoding a 401-amino acid protein of *Thermococcus profundus*, was cloned and expressed in *E. coli*. Recombinant α-amylase production was 155.5-fold higher than that in the wild strain (Lee et al., 1996). Another α-amylase gene (1383 bp encoding 461 amino acid residues) from a hyperthermophilic archaeon, *Pyrococcus* sp. KODl, was cloned and expressed in *E. coli*. The molecular mass of this mature enzyme is 49.456 kDa with 435 amino acid residues which displays < 40% homology to other amylases. The optimum temperature and pH for the enzyme activity are 90°C and pH 6.5. Ca^2+^ (2.0 mM) enhanced the thermostability of this enzyme (Tachibana et al., 1996). The recombinant α-amylase from *P. furiosus* is Ca^2+^-independent. This α-amylase is a liquefying enzyme with a specific activity of 3900 U mg^−1^ at 98°C. It was optimally active at pH 5.5–6.0 and 100°C with a half-life of 13 h at 98°C (Dong et al., 1997). Another α-amylase encoding gene from the extremely thermophilic archaeon, *T. hydrothermalis* was cloned and expressed in *E. coli*. The recombinant α-amylase possesses molecular characteristics similar to *Pyrococcus* species encoding a protein of 457 amino acids with a 22 amino acid putative signal peptide and a 435 amino acid mature protein (molecular mass 49.236 kDa). This recombinant α-amylase is optimally active at 75–85°C and pH 5.0–5.5 (Lévêque et al., 2000; Horvathova et al., 2006). Hyperthermophilic archaeon, *Pyrococcus woesei* also produces hyperthermophilic α-amylase, the gene encoding which has been cloned and expressed in the moderate halophile *Halomonas elongata*. The 14 kb protein-coding sequence, and biochemical properties of the expressed protein are identical to the α-amylase gene of the closely related *P. furiosus* (Frillingos et al., 2000). A maltogenic α-amylase encoding gene with 588 amino acids from thermophilic *Bacillus* sp. WPD 616 was cloned and expressed in *E*. *coli*, which showed optimum activity at pH 6.0 and 50°C (Liu et al., 2006). Sajedi et al. (2005) reported cloning and expression of α-amylase gene (1328 bp) from *Bacillus* sp. KR-8104 that encodes 440 amino acids without 20 amino acids of N and C termini in *E*. *coli*. A gene encoding a thermostable α-amylase with the temperature optima of 75°C from *G. thermoleovorans* YN cloned in Bluescript® II KS(+) vector in *E. coli* has been sequenced. The corresponding amino acid sequence showed 99% sequence similarity with the known α-amylases from different Bacilli and Geobacilli (Berekaa et al., 2007). Another α-amylase gene, Amy N, from *B. licheniformis* NH1 was also cloned and expressed in *E. coli* using pDEST17 expression system. This recombinant α-amylase exhibited higher thermostability at 85°C (T_1∕2_ 60 min) than the native amylase (8 min; Hmidet et al., 2008). In another study, the gene encoding α-amylase from *B. subtilis* PY22 was amplified by PCR and cloned into *Pichia pastoris* KM71H using the vector pPICZα, which allows methanol induced expression and secretion of the protein. The recombinant expression resulted in high levels of extracellular amylase production, as high as 22 mg/L in the shake flask culture supernatant (Karakas et al., 2010). A gene corresponding to thermo- and pH-stable maltogenic α-amylase of *G. caldoxylosilyticus* TK4 has been cloned into pET28a (+) vector and expressed in *E. coli* with 6xHis-tag at the N-terminus (Kolcuoglu et al., 2010). A gene encoding a hyperthermostable maltogenic α-amylase of *Staphylothermus marinus* (SMMA) was cloned and over expressed in *E*. *coli*. SMMA consisted of 696 amino acids with a predicted molecular weight of 82.5 kDa. The enzyme was active in acidic conditions (pH 3.5–5.0) with an optimal pH of 5.0, and was extremely thermostable with a temperature optimum of 100°C and a melting temperature of 109°C; both these favored starch conversion process (Li et al., 2010). The α-amylase encoding gene of an acidophile *B*. *acidicola* with N and C terminal truncation has been cloned in pET28a(+) and expressed in *E. coli* (Sharma and Satyanarayana, 2012). The 62 kDa recombinant α-amylase was optimally active at pH 4.0 and 60°C. Another raw starch digesting α-amylase gene (*amyBS-I*) with its signal peptide, from *B. subtilis* strain AS01a was cloned and expressed in *E. coli*. This recombinant enzyme was secreted extracellularly. The production was increased seven-fold by response surface optimization of culture conditions. This enzyme shows optimum activity at 70°C and pH 6.0. It is Ca^2+^ independent and was supplemented in bread dough for the amelioration of the bread quality as compared to the bread supplemented with commercial α-amylase (Roy et al., 2013). A gene encoding acidic, thermostable, and raw starch hydrolysing α-amylase was cloned from an extreme thermophile *G. thermoleovorans* and expressed (Mehta and Satyanarayana, 2013a). The ORF of 1650 bp encodes a 515 amino acid protein (Gt-amy) with a signal peptide of 34 amino acids at the N-terminus. The specific enzyme activity of recombinant Gt-amy is 1723 U mg^−1^ protein with a molecular weight of 59 kDa. It is optimally active at pH 5.0 and 80°C. Two other amylases with distinct properties from *G*. *thermoleovorans* have been cloned and expressed. One dimeric cyclodextrin-degrading maltogenic amylase with optimum temperature 80°C and pH activity between 5 and 9 (Mehta and Satyanarayana, 2013b), and the second raw starch hydrolysing thermostable α-amylase of 56 kDa with optimum activity at 60°C and pH 7.0 (Mehta and Satyanarayana, 2014). A gene encoding an α-amylase (Amy-E) from *Exiguobacterium* sp. SH3 was expressed in *E. coli* as a functional His-tagged protein of about 53 kDa with maximum activity at 30°C and pH 6.5. This enzyme retains 41% of its maximum activity at 0°C. This enzyme was biochemically characterized. It was halotolerant, and its activity was stimulated at high salt concentrations in the range of 1–5 M (Emampour et al., 2015). Another α-amylase gene from *B. amyloliquifaciens* was cloned into pMD18-T shuttle vector which was reconstructed to obtain vector pP43X for its heterologous expression in *B*. *subtilis* WB800. The recombinant purified enzyme had a specific activity of 5566 U/mg. Its productivity is 1.48-fold higher than the wild strain. The α-amylase gene encodes a protein of 514 amino acid residues with a predicted molecular weight of 58.4 kDa. The optimal conditions for its activity are pH 6.0 and 60°C. It was also biochemically characterized (Chen et al., 2015). The details of cloning and expression of α-amylase encoding genes are listed in Table 2.

**Table 2:** **Cloning and expression of α-amylase genes in heterologous hosts**.

	**Host**	**Gene length (bp)**	**Topt (°C) of recombinant α-amylase**	**pH optimum of recombinant α-amylase**	**References**
*Bacillus amyloliquifaciens*	*B*. *subtilis* WB800	1542	60	6.0	Chen et al., 2015
*B. licheniformis* NH1	*E. coli*	1539	90	5.0–10.0	Hmidet et al., 2008
*B. subtilis* WB800	*E. coli*	1545	60	6.0	Chen et al., 2015
*B. acidicola*	*E. coli*	1479	60	4.0	Sharma and Satyanarayana, 2012
*Bacillus* sp. KR-8104	*E. coli*	1328	75–80	4.0–6.0	Sajedi et al., 2005
*Bacillus* sp. WPD 616	*E. coli*	1764	50	6.0	Liu et al., 2006
*B. subtilis* DR8806	*E. coli*	1545	70	5.0	Emtenani et al., 2015
*B. subtilis* PY22	*Pichia pastoris*	1960	60	7.0	Karakas et al., 2010
*B. subtilis* strain AS01a	*E. coli*	1977	70	6.0	Roy et al., 2013
*Exiguobacterium* sp. SH3	*E. coli*	1443	30	6.5	Emampour et al., 2015
*Geobacillus thermoleovorans*	*E. coli*	1650	80	5.0	Mehta and Satyanarayana, 2013a
*G. thermoleovorans*	*E. coli*	1767	80	5.0–9.0	Mehta and Satyanarayana, 2013b
*G. thermoleovorans*	*E. coli*	1542	60	7.0	Mehta and Satyanarayana, 2014
*G. thermoleovorans* YN	*E. coli*	1649	75	–	Berekaa et al., 2007
*G. caldoxylosilyticus* TK4	*E. coli*	1740	50	7.0	Kolcuoglu et al., 2010
*Halomonas meridiana*	*E. coli*	1371	–	–	Coronado et al., 2000b
*Pseudoalteromonas* sp. MY-1	*E. coli*	2007	40	7.0	Tao et al., 2008
*Pyrococcus furiosus*	*E. coli*	1380	100	5.5–6.0	Dong et al., 1997
*Pyrococcus* sp. KODl	*E. coli*	1383	90	6.5	Tachibana et al., 1996
*P. woesei*	*Halomonas elongata*	1400	90–100	5.5–6.0	Frillingos et al., 2000
*Staphylothermus marinus*	*E. coli*	2088	100	5.0	Li et al., 2010
*Streptomyces lividans* TK24	*E. coli*	1719	–	–	Yin et al., 1997
*Thermobifida fusca*	*Pichia pastoris*	1815	60	7.0	Yang et al., 2010
*Thermococcus hydrothermalis*	*E. coli*	1371	75–85	5.0–5.5	Lévêque et al., 2000; Horvathova et al., 2006
*T. profundus*	*E. coli*	1203	80	4.0–5.0	Lee et al., 1996
*Thermoplasma volcanicum* GSS1	*E. coli*	1872	80	–	Kim et al., 2007
*Thermotoga maritima* MSB8	*E. coli*	1269	70	7.0	Lim et al., 2003

## Protein engineering of α-amylases and molecular methods for their amelioration

Since several decades, a wide array of techniques have been developed allowing engineering of the enzyme properties. Directed evolution and site-directed mutagenesis are powerful tools for engineering enzymes, to improve their functions and to alter their properties like activity, selectivity, substrate specificity, stability, and solubility (Rubin-Pitel and Zhao, 2006). Thermostability, activity in acidic range and Ca^2+^-independence of α-amylases are desirable for their use in the starch saccharification process, and activity in alkaline range and oxidative stability is a prerequisite for its applicability in detergent industry. These and several other properties of α-amylases have been improved by various methods such as site-directed mutagenesis or directed evolution approach, which are described below.

### Protein engineering of bacterial and archaeal α-amylases to increase thermostability

Many factors contribute to the stability of thermostable proteins including the presence of hydrogen bonds, electrostatic interactions, salt bridges, hydrophobic interactions, disulfide bonds, reduced entropy of unfolding, oligomerization, increased occurrence of proline residues, and others (Salminen et al., 1996; Russell et al., 1997; Vogt et al., 1997; Mehta and Satyanarayana, 2013b, 2015). Any of these properties of an enzyme can be modified by using methods such as site-directed mutagenesis, directed evolution, deletion mutation, and others for increasing its thermostability. Some examples of increasing thermostability of α-amylases are explained as follows. The first determinants of thermostability in bacterial α-amylases were identified by comparison of amino acid sequences of α-amylases of *B. licheniformis* (BLA) and *B. amyloliquifaciens* (BAA). Three stabilizing mutations in BAA were proposed, which caused a significant and additive thermostabilization of BAA; deletion of R176 and G177, and the substitution of K269A (Suzuki et al., 1989). This R176-G177 deletion showed similar effects on the thermostability of other α-amylases as well (Igarashi et al., 1998; Bisgaad-Frantzen et al., 1999). Conrad et al. (1995) separately determined four regions of particular importance for the thermostability of BLA, namely 34–76, 112–142, 174–179, and 263–276. Two of these coincide with the mutations identified earlier by Suzuki et al. (1989). BLA has been further mutated to withstand high temperature and acidic pH. Two positions 133 and 209 have been identified as important for its thermostability (Declerck et al., 1990; Joyet et al., 1992). Twelve different amino acids substitutions were made at H133 by a tRNA suppressor method, and H133Y substitution improved the stability the most (Declerck et al., 1990). Random mutagenesis and screening approach was used to create A209V substitution. The stabilizing effect of this mutation was an additive with the H133Y mutation, and the half-life of the double mutant was 10-fold higher at 90°C (Joyet et al., 1992). All naturally occurring amino acids were introduced in each of these two positions (Declerck et al., 1995). Of these, H133I substitution had stronger effect on thermostabilization than the previous substitution. Random mutagenesis and screening have also been used to identify M15T and N188S as stabilizing mutations of BLA (Shaw et al., 1999). A 23-fold enhancement in stability was achieved at pH 5.0 and 83°C in the presence of 5 mM CaCl_2_ by combining these mutations with H133Y/A209V. Shaw et al. (1999) also predicted a stabilizing effect for additional substitutions: A33S, A52S, N96Q, S148N, and A379S. Four rounds of DNA shuffling and subsequent recombination produced a variant from *Thermus* sp. IM6051 maltogenic amylase with a 15°C increase in optimum temperature as compared to wild-type (Kim et al., 2003). The half-life of this variant was 172 min at 80°C as compared to the wild type, which was completely inactivated at this temperature. After three rounds of DNA shuffling of *B. thermalkalophilus* ET2 maltogenic amylase, followed by recombination of selected mutations, variants with optimal reaction temperatures 10°C higher than the native enzyme was achieved. One of the variants carrying mutations displayed a 20-fold longer half-life than wild-type at 78°C (Tang et al., 2006). To improve the performance of a maltogenic amylase (Novamyl) as an antistaling agent for breads made at low pH, two epPCR libraries of Novamyl were constructed. These libraries were screened for improved thermal stability at 80°C and activity at pH 4.3 for 25 min. A triple mutant was better than the wild type for antistaling activity in bread made at pH 4.3 and exhibited a 10°C increase in melting temperature at pH 4.0 as compared to the wild type (Jones et al., 2008). The effect of deletion was investigated on the half-life of *Bacillus* sp. TS-23 α-amylase. An increased half-life at 70°C was observed for the mutant enzyme suggesting that Arg210-Ser211 deletion leads to a conformational change of the enzyme (Lin et al., 2008). Site-directed mutagenesis at Asn-75, Ser-76, and His-77 calcium binding sites of α-amylase from *B. megaterium* WHO resulted in increased thermostability. In the presence of calcium, conversion of His-77 to Glu resulted in a four-fold enhancement in enzyme half-life and a 9°C upward shift in *T*_50_, which was observed in comparison with the wild type. The H77E mutant was most stable with increased affinity for calcium ion and 5°C higher optimum temperature than the wild type (Ghollasi et al., 2013). The α-amylase (Ba-amy) of *Bacillus acidicola* was fused with gene fragments encoding partial N- and C-terminal region of thermostable α-amylase gene of *G. thermoleovorans*. The chimeric enzyme (Ba-Gt-amy) expressed in *E. coli* showed higher catalytic efficiency and thermostability than Ba-amy. The melting temperature (*T*_m_) of Ba-Gt-amy (73.8°C) was also higher than Ba-amy (62°C). Circular dichroism (CD) spectra also revealed the stability of the chimeric enzyme (Parashar and Satyanarayana, 2016).

### Protein engineering of bacterial and archaeal α-amylases for alteration of pH optima and stability

Site directed mutagenesis has also been employed for altering the optimum pH and increasing acid/basic stability of an enzyme. This method reduces the time and cost spent on screening libraries for improved variants. Saturated mutagenesis of the starch-binding domain of the α-amylase from *B*. *licheniformis* followed by selection for starch binding at low pH yielded a double mutant with an improved starch hydrolysis ratio at pH 4.5 as compared to the native protein (Verhaert et al., 2002). Site directed mutagenesis of soybean α-amylase led to the removal of hydrogen bond networks around the catalytic base residue (E380) of the enzyme (Hirata et al., 2004), raising the optimum from pH 5.4 to a more neutral pH range between 6.0 and 6.6. Richardson et al. (2002) used activity and sequence based screening to single and multi-organism DNA libraries from various environments to identify three different α-amylases with one or more aspects of the necessary phenotype: temperature stability, pH optimum, and lowered requirement of Ca^2+^. The genes encoding amylases with these properties were used as parental sequences for DNA shuffling in order to combine the best aspects of the three enzyme phenotypes. The two best chimeric α-amylases found by high throughput screening had 40-fold longer half-life for activity in the absence of Ca^2+^ ions at 90°C and pH 4.5 as compared to the most stable wild-type parent. The α-amylase from *B. amyloliquefaciens* with optimal activity at pH 6.0 was engineered by error prone PCR (epPCR) to produce a variant having optimal activity at alkaline pH for the detergent industry. The screening of an epPCR library for amylase activity at pH 7.0 and pH 10.0 yielded 26 variants with a very high activity. Sixteen of the selected variants were then randomly recombined by DNA shuffling and screened for increased activity at pH 10. The best mutant displayed a five-fold higher activity at pH 10 than the wild type (Bessler et al., 2003). Liu et al. (2008) observed that the mutations at two crucial positions Leu_134_ and Ser_320_ together affected the acid resistance of the α-amylase of *B. licheniformis* CICC 10181. Directed evolution was used to increase acid stability. In another example, protein stability and catalytic efficiency of α-amylase from *B. subtilis* was increased under acidic conditions by site-directed mutagenesis. Based on the three dimensional structure model analysis, four basic histidine (His) residues His^222^, His^275^, His^293^, and His^310^ in the catalytic domain were found to be important and single, double as well as triple mutants were constructed at these sites. The acidic stability of enzyme was significantly enhanced after mutation, and 45–92% of initial activity of mutants was retained after incubation at pH 4.5 and 25°C for 24 h as compared to the wild-type (39.5%). The catalytic efficiency for each active mutant was also much higher than that of wild-type at low pH. Due to increase in the hydrogen bonds and salt bridges after mutation, an obvious shift of the basic limb toward acidity was observed. These changes around the catalytic domain contributed to the significantly improved protein stability and catalytic efficiency at low pH (Yang et al., 2013).

### Protein engineering of bacterial and archaeal α-amylases for increasing oxidative stability

Oxidation also has a demonstrated negative effect on the stability of α-amylases. Cysteine 362 was identified as the oxidation prone residue in BStA (Tomazic and Klibanov, 1988; Brosnan et al., 1992), and methionine 197, which is situated close to the active site, has been shown to be responsible for the inactivation of BLA (Borchert et al., 1995). The introduction of any non-sulfur-containing amino acid at position 197 was shown to greatly reduce the oxidation sensitivity of BLA. The specific activity was, however, very much dependent on the introduced side chain, with an apparent preference for the smaller side chains (Borchert et al., 1995). *Bacillus* sp. TS-23 α-amylase was truncated at both N and C termini, and further mutated to increase thermal and oxidative stability. Met231 was mutated to leucine and 483th codon of this enzyme was mutated to stop codon i.e., TAA by site-directed mutagenesis. The resultant engineered enzyme showed higher T_1∕2_ at 70°C and showed compatibility to detergents (oxidative stability; Chi et al., 2010). In another example, oxidative stability of α-amylase (AmyC) from *Thermotoga maritima* was improved by site-directed mutagenesis. In this, methionine residues at positions 43 and 44, 55, and 62 were mutated to alanine, which is oxidative-resistant. The M55A mutant showed 50% residual activity in the presence of H_2_O_2_, while the wild-type α-amylase was inactive (Ozturk et al., 2013).

### Protein engineering of bacterial and archaeal α-amylases for the alteration of transglycosylation/hydrolysis ratio

Product re-hydrolyzation is a problem during maltooligosaccharide synthesis using maltogenic amylase. Maltogenic amylase (MAG1) from *Bacillus lehensis* G1 was mutated using a structure-guided protein engineering approach to decrease the hydrolysis activity of the enzyme. W359F, Y377F, and M375I mutations reduced steric interference, altered the subsite occupation and increased the internal flexibility to accommodate longer donor/acceptor molecules for transglycosylation, which resulted in an increase in the transglycosylation to hydrolysis ratio of up to 4.0-fold (Manas et al., 2015). Maltogenic amylases also use donor and acceptor sugar molecules to form α-1,4 or 1,6 glycosidic linkages between them. This process, called transglycosylation, is responsible for formation of undesirable maltotriose in the high maltose syrups. Maltogenic amylases with high hydrolytic activity than transglycosylation activity are used to decrease the maltotriose content of high maltose syrups. Maltogenic amylase from *B. stearothermophilus* was engineered by site-directed mutagenesis of W177 to decrease its transglycosylation activity. The mutant W177S exhibited notable increased maltose production with a minimum amount of maltotriose under industrial conditions (Sun et al., 2016). Role of His and Glu in the catalytic activity of *B. licheniformis* α-amylase (BLA) was determined by performing site-directed mutagenesis at His235. It was observed that the mutant enzyme (H235E) displayed high substrate transglycosylation activity, while the wild-type BLA exhibited high hydrolysis activity exclusively. This shows that Glu235 located on a wide open groove near subsite +1 is most probably involved in transglycosylation and may recognize and stabilize the non-reducing end glucose of the acceptor molecule (Tran et al., 2014)

## Sequence and structural diversity of α-amylases

### Conserved regions of GH-13 family

**Figure 1:**
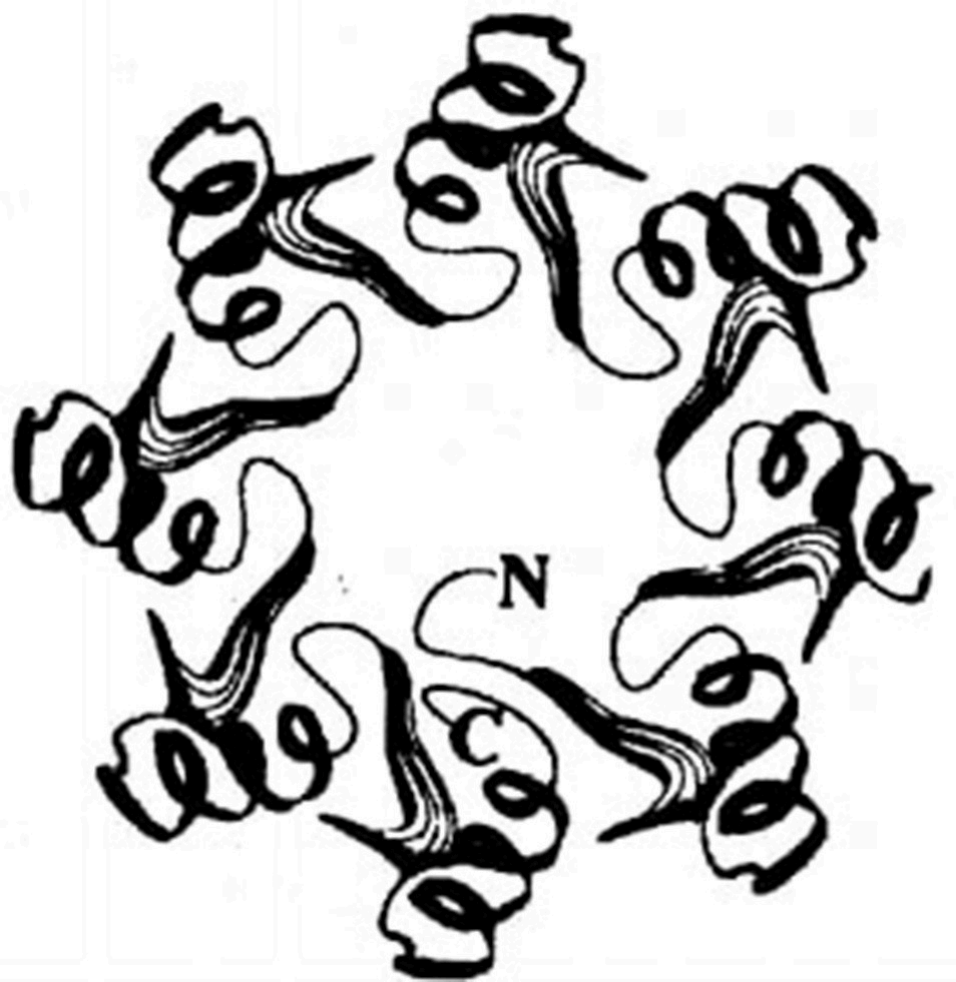
**Schematic representation of the (β/α)_**8**_ barrel (TIM barrel)**.

According to the Carbohydrate-Active enZYmes (CAZY) database, the α-amylases are grouped with different kinds of glycosyl hydrolases in GH-13 family, also known as α-amylase family (Henrissat, 1991). This family contains the enzymes with the following characteristics. They act on α-glycosidic bonds and hydrolyze it to produce α-anomeric mono-or oligosaccharides (hydrolysis) or form α-1,4 or 1,6 glycosidic linkages (transglycosylation), or show both activities. They possess a (β/α)_8_ or TIM barrel structure that contains the catalytic site residues (Figure 1). Their primary sequences have seven highly conserved regions with catalytic amino acids and some amino acids that are essential for the stability of the topology of conserved TIM barrel (Kuriki and Imanaka, 1999). The first four conserved regions (I to IV) are found in the TIM-barrel on β-strands 3, 4, and 5 and in the loop connecting β-strand 7 to α-helix 7. Amino acid residues corresponding to region I are found in the C-terminal end of the third β-strand of the TIM barrel (β3). This region includes the three conserved amino acids Asp 100, Asn104, and His105 (BLA numbering). Asp100 is essential for the active site integrity because it forms hydrogen bonding with Arg229. Arg229 is a completely conserved residue. This is present within hydrogen bond distance of the catalytic nucleophile Asp231 and the proton donor Glu26. Asn104 coordinates the conserved calcium ion between the A and B domains and does not directly stabilize the active site (Boel et al., 1990; MacHius et al., 1995, 1998). His105 stabilizes the interaction between the C-terminal of β3 and the rest of TIM barrel by hydrogen bonding to Asn104 and to the backbone oxygen of Tyr56 (BLA numbering), which is situated in the loop connecting β2 to α2. Region II is positioned in β4 and has the catalytic nucleophile Asp231 and the invariant residue Arg229. These two residues are indispensable for catalytic activity and are found in all α-amylases (Svensson, 1994). Lys234 and His235 are also present in this region, which form part of subsite +2. These amino acids bind the reducing end of the glucose chain in the substrate-binding cleft (Svensson, 1994). Region III includes the conserved residue and catalytic proton donor Glu261, which lies in the C-terminal part of the fifth L-strand of the TIM-barrel. The residues forming region IV are situated in the loop connecting L7 to K7 and protect the active site from the solvent. This region has fully conserved residue Asp328, which is postulated to be involved in substrate binding, substrate distortion and in elevating the pKa of Glu261. There is a strong preference for His, Phe, Val and Asn are often observed at positions 327, 323, 324, and 326, respectively (Klein et al., 1992; Knegtel et al., 1995; Strokopytov et al., 1995). The number of residues between the conserved Gly and Pro of the region VI differentiates between an α-amylase (7 residues) and a CGTase (8 residues; Janecek et al., 1995). Region VII does not contain conserved residues, but it usually starts with Gly followed by proline at i + 2 position.

### GH-13 vs. GH-57 family: a comparison with respect to archaeal α-amylases

**Figure 2:**
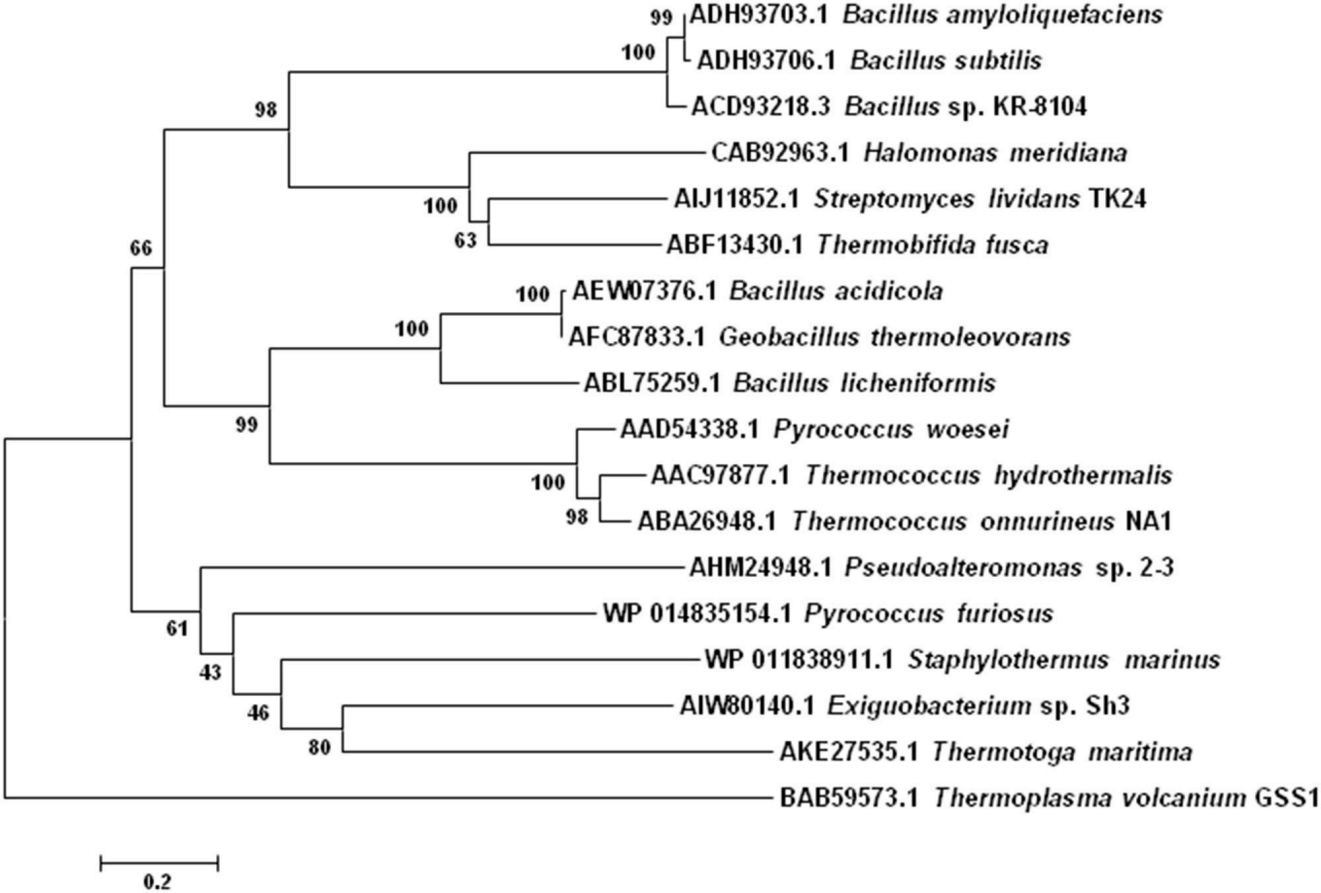
**Phylogenetic tree showing relationships among various bacterial and archaeal α-amylases**. The sequences of α-amylases from representative species of different bacterial and archaeal genera in FASTA format have been obtained from NCBI database. Phylogenetic tree was constructed using MEGA 4 software. The accession number of each bacterial and archaeal α-amylase sequence is mentioned that reveals the exact source of the sequence of representative species.

Comparison of the *T. hydrothermalis* α-amylase sequence with those of 21 other bacterial and archaeal α-amylases, which were representatives of more than 100 known α-amylase sequences, revealed that this enzyme as well as the other Thermococcales secreted α-amylases, contain most of the β-strands and the pentapeptide stretch located near the C-terminus of loop β3 → α3 that are present in family GH-13 (Janecek, 1997; Janecek et al., 1999). This indicates that α-amylase secreted by the members of Thermococcales belong to family GH-13. However, α-amylase from *P. furiosus* does not contain these conserved regions, so it belongs to GH-57 family instead. It was also showed that the α-amylase from the methanogenic archaeon *Methanococcus jannaschii* contains features of both glycosl hydrolase families, which indicates that these two families are either derived from a distant common ancestor or that one evolved from the other (Janecek, 1998). Also, the conserved regions of Thermococcale α-amylases show some characteristic sequence features (Janecek et al., 1999). A tryptophan is present in all the archaeal enzymes in the β4 strand. Moreover, a histidine, which has been suggested to be responsible for substrate binding, is replaced by glycine in archaeal enzymes (Svensson, 1994). It was suggested that the tryptophan found in β4 strand might be involved in catalysis instead of the missing histidine (Lee et al., 1996). Phylogenetic analysis revealed that archaeal α-amylases are closely related to plant α-amylases (Janecek et al., 1999). It also suggested that archaeal α-amylases are closer to the liquefying α-amylases, than to the saccharifying ones, which also agrees with the nature of the products of starch hydrolysis by the archaeal enzymes (Janecek et al., 1999). The phylogenetic analysis of various archaeal and bacterial α-amylases is shown in Figure 2.

### Domain organization in α-amylases

**Figure 3:**
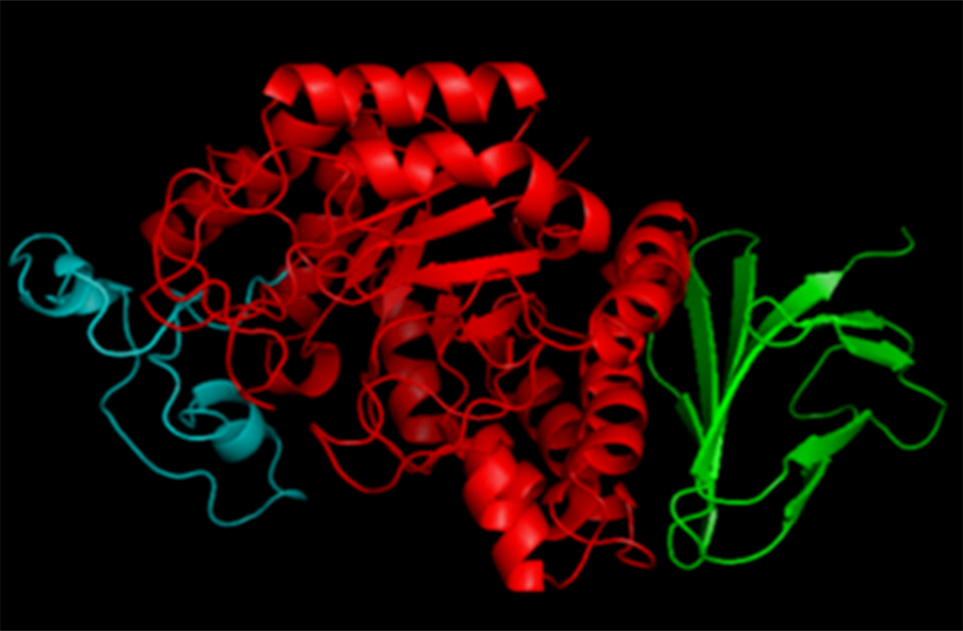
**Domain organization in α-amylases**. Domain A is shown in red, domain B in cyan, and domain C in green. The structure of this amylase is prepared using homology modeling. The sequence information for homology modeling is obtained from NCBI database for Gt-amyI amylase (Mehta and Satyanarayana, 2013a).

The structure of α-amylase, in general, consists of a polypeptide chain folded into three domains called A, B, and C (Figure 3). Domain A is the catalytic domain, which consists of N-terminal (β/α)_8_ barrel. This consists of a highly symmetrical fold which includes eight parallel β-strands organized in a barrel with a border of eight α-helices. This (β/α)_8_ barrel was first observed in chicken muscle triosephosphate isomerase (TIM), which (Banner et al., 1975), and hence, it is called TIM barrel (Figure 1). It is present in all the members of α-amylase family (Banner et al., 1975). The overhang between the third β-strand and the third α-helix of the TIM barrel forms Domain B. Domain B has an irregular β-sheet structure, forms a large part of the substrate binding cleft and varies considerably in structure among different α-amylases. This domain is presumed to play a role in the substrate specificity differences observed in the α-amylases (Janecek et al., 1997). Domain C constitutes the C-terminal part of the sequence and is composed of β-sandwich structure (MacGregor, 1988). Both domains B and C are located at opposite sides of the TIM barrel. Mehta and Satyanarayana (2014) have recently attributed the role of raw starch binding to the C domain of *G*. *thermoleovorans* α-amylase (Gt-amyII). Whether this is generalized for all α-amylases or only for this particular enzyme is not yet clear. In this enzyme, two tryptophan residues, W204 and W205, corresponding to W201/W202 of BaqA (*B*. *aquimaris* α-amylase) and W200/W201 of *Anoxybacillus* sp. α-amylase are present (Chai et al., 2012; Mok et al., 2013; Puspasari et al., 2013), which precedes β4 strand covering the catalytic nucleophile. These two tryptophan residues have been postulated to bind sugar residues of the raw starch in raw starch degrading α-amylase Baq via stacking interactions (Puspasari et al., 2013). Detailed structural analysis reveals that in GTA and Gt-amyII, W205 is not solvent accessible and is buried, in comparison to Baq, where both tryptophans are accessible to solvent (Mok et al., 2013). The domain C has also been shown to play a role in raw starch binding in barley α-amylase (Robert et al., 2003). In barley α-amylase, a “pair of sugar tongs” site in the domain C, formed by Ser378 and Tyr380, had been shown to function in starch recognition and binding (Bozonnet et al., 2007). It may be possible that a similar site is present in Gt-amyII (Mehta and Satyanarayana, 2014).

In the maltogenic amylases, the C-domain is followed by a D-domain, the function of which is also unknown (Nielsen and Borchert, 2000). A recent investigation by Tan et al. (2008) on the crystal structure of a thermostable α-amylase Amy B from *H. orenii* showed that it also has an additional N-terminal domain called N domain that forms a large groove, the N–C groove, in addition to the typical domain organization of GH-13 family (i.e., A, B, and C). It was shown that the N domain has been shown to increase the binding of enzyme to the raw starch. This N-domain is also found in maltogenic amylases. It has been shown to be responsible for thermostabilization via oligomerization and substrate affinity modifications in maltogenic amylases of *G. thermoleovorans* and *Thermus* sp. (Kim et al., 2001; Mehta and Satyanarayana, 2013b).

Approximately 10% of the amylolytic enzymes possess a separate domain for binding to raw starch. Besides α-amylases, the starch binding function has been reported from some glycoside hydrolases, cyclodextrin glucanotransferases, and acarviose transferases from glycoside hydrolase family GH13, β-amylases from GH14, and glucoamylases from GH15. SBDs are classified under CBMs (carbohydrate binding modules), which have been classified into 67 families (http://www.cazy.org/Carbohydrate-Binding-Modules.html) on the basis of amino acid sequence similarity. Starch-binding domain belongs to seven different families of CBMs (CBM20, CBM21, CBM 25, CBM26, CBM34, CBM41, and CBM48). α-Amylase SBDs are majorly classified in CBM20 and CBM25 families. Although large number of bacterial, archaeal as well as fungal SBD sequences are available at CAZy database; only a few α-amylase SBDs have been characterized and well described (Iefuji et al., 1996; Sumitani et al., 2000; Guillén et al., 2007; Yamaguchi et al., 2012). Florencio et al. (2000) and Morlon-Guyot et al. (2001) reported the presence of starch binding domains (SBDs) in three α-amylases from Lactobacilli. The genes encoding the α-amylases have been sequenced (Giraud and Cuny, 1997; Morlon-Guyot et al., 2001), and the amino acid sequence analysis revealed that the C-terminal possesses SBD belonging to CBM 26 family formed by direct tandem repeat units; four modules have been reported in *Lactobacillus plantarum* and *L. manihotivorans* α-amylases and five in that from *L. amylovorus* enzyme. The enhanced affinity in the SBD of the maltohexaose-forming amylase from *B. halodurans* is due to the simultaneous interaction of the two tandem CBMs present in the enzyme (one from family CBM 25 and the other from family CBM 26). Structural features of SBDs indicate that they belong to type B glycan chain-binding CBMs. The members of this class comprise several subsites that are able to accommodate individual sugar units of the polymeric ligands. These CBMs show higher affinity toward oligosaccharides and higher polymers, and show negligible interaction with oligosaccharides with a degree of polymerization (DP) of three or less. These are called as “chain binders.” According to the Boraston's classification of CBMs based on the kind of fold (Boraston et al., 2004; Hashimoto, 2006), most of the SBDs are classified in this fold family 1 of CBMs.

### Ca^2+^ and CL^−^ binding sites in α-amylase structure

The most intensively studied bacterial and archaeal α-amylases for which 3D structures have been resolved (Kim et al., 1999) include: *Alteromonas haloplanctis* (AHA), *B*. *subtilis* (BSUA), *B*. *amyloliquefaciens* (BAA), *B*. *licheniformis* (BLA), *P. woesei*/*furiosus* (PWA/PFA; Koradi et al., 1996). These α-amylases vary considerably with respect to the properties. In general, all known α-amylases contain a conserved calcium ion, which is present at the interface between domains A and B (Boel et al., 1990; MacHius et al., 1995; Linden et al., 2003). This calcium ion is indispensable for its stability and activity of α-amylases (MacHius et al., 1998). If the calcium is removed experimentally, the α-amylase loses its catalytic properties, whereas its restoration recovers the activity. Calcium ion is positioned far away from the active site to contribute directly in catalysis. Therefore, the role of calcium ion has been suggested to be structural (Hsiu et al., 1964; Larson et al., 1994). Calcium ion also plays a role in the stabilization of thermostability of α-amylases. This can be explained by the fact that salting out of hydrophobic residues by calcium ion in the protein structure leads to the formation of a compact structure (Buisson et al., 1987). Many α-amylases contain a single calcium ion, while α-amylases from *Bacillus* spp. contain three or four calcium ions and one sodium ion and a calcium—sodium—calcium metal triad, which bridges between domains A and B (Feller et al., 1999; Linden et al., 2003). This metal triad contributes to maintaining the conformational stability of protein as well as for resisting thermal inactivation of the enzyme (MacGregor, 1988; Khajeh et al., 2001; Goyal et al., 2005). There are conserved Ca^2+^ binding sites to which calcium ion bind in most of the α-amylases. A new type of *Bacillus* α-amylase (Amy K38), a calcium-free amylase has been reported, in which two sodium ions retain the structure and function of the enzyme, instead of calcium ions (Lee et al., 2006). Besides this, there are other α-amylases that are Ca^2+^-independent. These include enzymes from *P*. *furiosus, Thermus* sp., *G. thermoleovorans* etc (Koch et al., 1990; Malhotra et al., 2000; Nonaka et al., 2003; Prakash and Jaiswal, 2010; Mehta and Satyanarayana, 2013b, 2014). Ca^2+^, which stimulates most of the amylases, inhibits another α-amylase in *G*. *thermoleovorans* (Mehta and Satyanarayana, 2013a), and in *B*. *coagulans* B 49 (Babu and Satyanarayana, 1993). Besides the primary Ca^2+^ binding sites, calcium ions also bind to the secondary sites, which involves catalytic residues (Asp and Glu) located at the bottom of substrate binding cleft. The presence of these secondary Ca^2+^ binding sites at catalytic sites explains inhibition of α-amylase by Ca^2+^ at higher concentration (MacHius et al., 1995; Mehta and Satyanarayana, 2013a). Many α-amylases also contain a chloride ion in the active site, which enhances the catalytic efficiency of the enzyme (Levitsky and Steer, 1974; Feller et al., 1996). Although majorly found in mammalian α-amylases (Larson et al., 1994; Brayer et al., 1995; Ramasubbu et al., 1996), a chloride ion has also been reported in a α-amylase (AHA) from the psychrophilic bacterium, *A. haloplanctis* (Aghajari et al., 1998). Chloride binding increases the affinity of the enzyme for the calcium ion, therefore it is much likely that binding of chloride ion also induces conformational changes around the active site (Levitsky and Steer, 1974).

### Active site cleft of α-amylases

In total, three steps are involved in the catalytic mechanism for retaining glycosyl hydrolases (Davies and Henrissat, 1995). Firstly, the glycosidic oxygen is protonated by the proton donor (Glu261). This is followed by a nucleophilic attack on the C1 of the sugar residue in subsite-1 by Asp231 (BLA numbering; Nielsen et al., 1999). Once the glycon part of the substrate leaves, a water molecule is activated presumably by the deprotonated Glu261. This water molecule hydrolyses the covalent bond between the nucleophilic oxygen and the Cl of the sugar residue in subsite-1, thereby completing the catalytic cycle (Nielsen et al., 1999).

**Figure 4:**
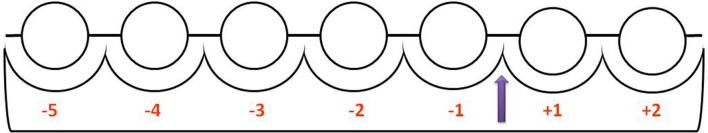
**Active site subsite nomenclature for glycosyl hydrolases**.

At the C-terminus of the β-strands in the TIM barrel, in the interface between domain A and domain B lies the active site cleft. The substrate binding cleft can accommodate from four to ten glucose units as revealed by the X-ray structures of α-amylases complexed with the α-amylase inhibitor acarbose. Certain of the amino acid residues of α-amylase binds to each glucose unit, which is said to constitute the binding subsite for that glucose unit. Subsite nomenclature was defined by Davies et al. (1997). The location of scissile bond defines the numbering of the subsites, with negative subsite numbers on the non-reducing side of the scissile bond, and positive subsite numbers on the reducing end (Figure 4). In the α-amylases the number of subsites present on the reducing end of the scissile bond are two or three (subsites +1, +2, and +3), while those on the non-reducing side of scissile bond varies between two and seven (MacGregor, 1988; Brzozowski et al., 2000). The X-ray structures of α-amylases complexed with acarbose shows that acarbose occupies subsites −1 to +4. Density for more than one sugar units is also observed in these X-ray structures in addition to the density for the four sugar units from acarbose (Brzozowski and Davies, 1997; Dauter et al., 1999). A study on a chimaeric *Bacillus* α-amylase (BA2) revealed that the extra density originates from a transglycosylation event that produces longer chained sugar molecules, which span the subsites −7 to +3, in comparison to an active site mutant of BA2, in which acarbose molecule spans the subsites −7 to −4. This observation suggests that the catalytic activity of the α-amylases is necessary for converting acarbose into a more potent longer-chained inhibitor by transglycosylation (Brzozowski et al., 2000).

## Applications of α-amylase

**Figure 5:**
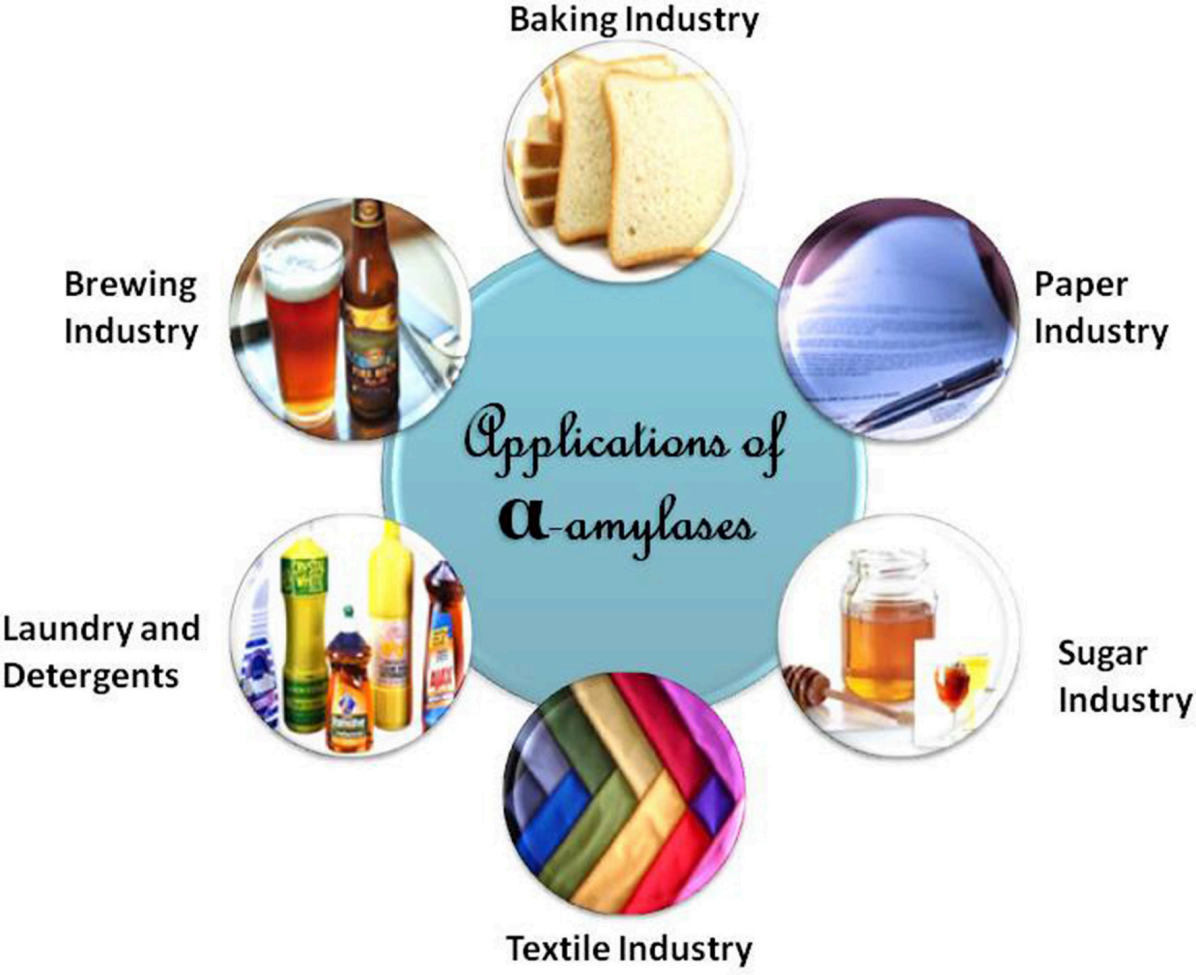
**Applications of α-amylases**.

**Figure 6:**
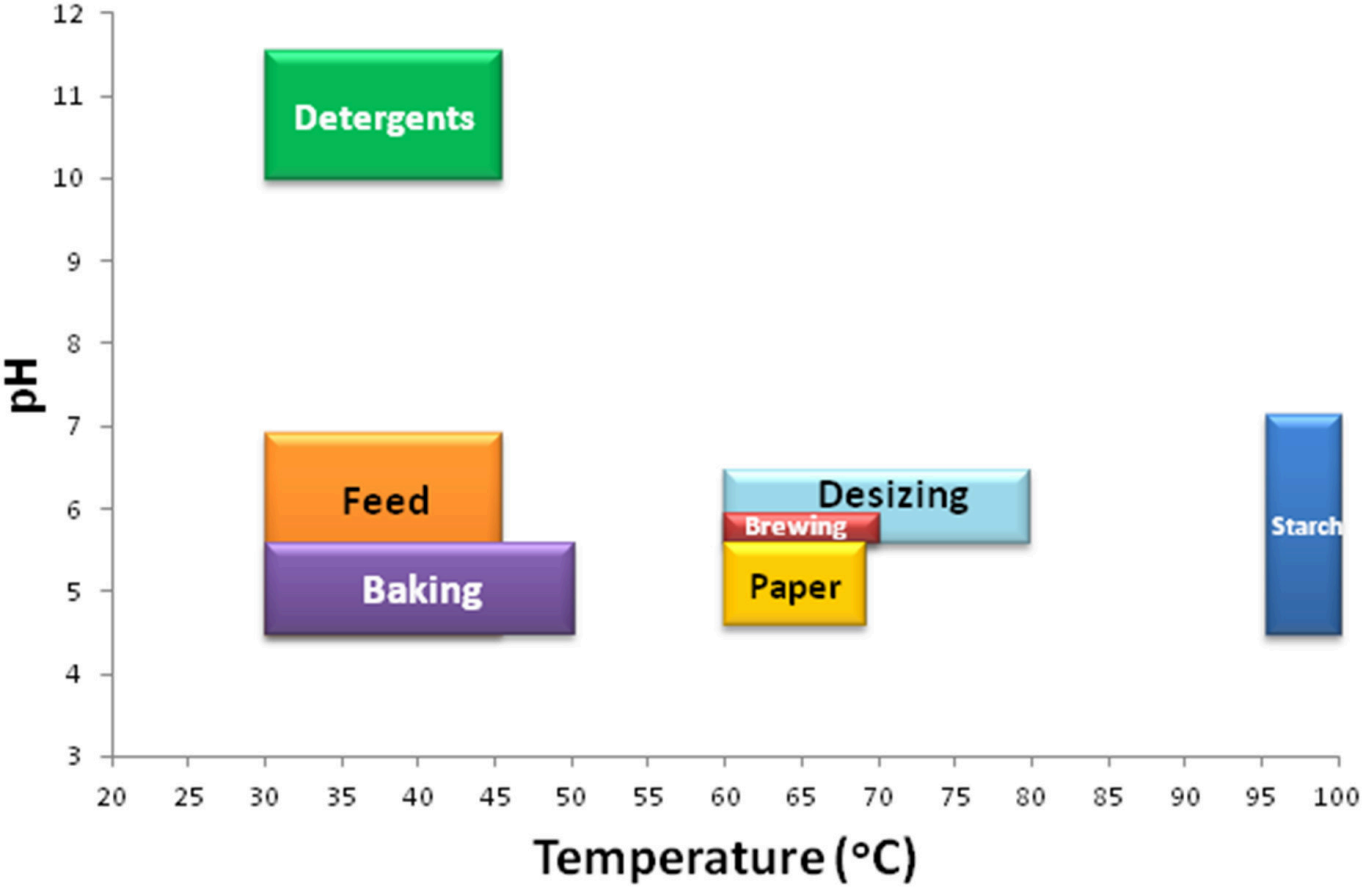
**Characteristics of α-amylases for specific applications**. A bar graph is plotted using the range of temperature and pH requirements of α-amylases to be used for different industrial applications. The temperature/pH range of α-amylases to be used in detergent industry, feed, baking, desizing, brewing, paper industry, and starch saccharification are 30–45°C/10.0–11.5, 30–45°C/4.5–7.0, 30–50°C/4.5–5.5, 60–80°C/5.5–6.5, 60–70°C/5.5–6.0, 60–70°C/4.5–5.5, 95–100°C/4.5–7.0, respectively.

α-Amylases can be used at industrial level in sugar, textile, baking, paper, and brewing industries (Figure 5). Every application requires an α-amylase with specific characteristics (Figure 6). Different commercially available α-amylases with varying properties are used for different applications. A list of commercially available α-amylases is presented in Table 3. The foremost applicability of α-amylases in sugar industry is in the formation of high fructose corn syrups (HFCS), which are used in huge quantities in the beverage industry as sweeteners for soft drinks. Starch saccharification is required for the production of high fructose corn syrups (HFCS; Guzman-Maldonadao and Paredes-Lopez, 1995; Crabb and Mitchinson, 1997). During liquefaction, starch granules are gelatinized in a jet cooker at 105–110°C for 5 min (pH 5.8–6.5) and then partially hydrolyzed using thermostable α-amylase at 95°C for 2–3 h (Vieille and Zeikus, 2001). After liquefaction, the pH is adjusted to 4.2–5.0 and the temperature is lowered to 55–60°C for the saccharification step, which produces dextrins. The amylolytic enzymes that produce specific malto-oligosaccharides in high yields from starch have gained significant attention. Such enzymes are widely used in the food, chemical and pharmaceutical industries (Nigam and Singh, 1995). Although maltogenic α-amylases that yield 53–80% maltose have been reported, their industrial potential is limited because of their moderate thermostability, Ca^2+^ requirement and transglycosylation property. The use of Ca^2+^-independent enzymes in starch hydrolysis eliminates the addition of Ca^2+^ in starch liquefaction and its subsequent removal by ion exchangers from the product streams (van der Maarel et al., 2002). α-Amylases from several microbes such as *G. thermoleovorans, Bacillus megaterium* VUMB109, *P. furiosus*, and others have been reported to possess most of the desired properties, which enable them to produce high yields of maltooligosaccharides and maltose (Rao and Satyanarayana, 2007b; Jana et al., 2013; Cuong et al., 2016). Some raw starch degrading α-amylases have also been reported which can act at the native pH of starch, thereby avoiding the pH adjustment step in the starch saccharification process. Hydrolysis rates of corn and wheat starches at 60°C were 65.5 and 70.3% with 10.0% starch slurry (Mehta and Satyanarayana, 2013a). Hydrolysis rates of 60 and 37% of 1% raw wheat and corn starches in 4 h has been recorded with 0.07 U mg^−1^ starch of *B. licheniformis* ATCC 9945a α-amylase (Bozic et al., 2011). The α-amylase of *Alicyclobacillus* at 0.5 U mg^−^1 starch hydrolyzed 52.0% of 1.0% corn starch in 2 h (Bai et al., 2012). While *G. thermoleovorans* subsp. *stromboliensis* α- amylase at 500 U g^−1^ starch, hydrolyzed 50.0% of the corn starch (20%) in 12 h (Finore et al., 2011).

**Table 3:** **Commercially available bacterial α-amylases**.

**Commercial name of α-amylase**	**Manufacturer**	**Producer microorganism**	**Application**
Amzyme TX	Parchem^7^	*Bacillus amyloliquifaciens*	Foods and feeds
Aquazym 120l	Novo Nordisk, Denmark^5^	–	Desizing of textiles
Aquazym Ultra 250l	Novo Nordisk, Denmark^5^	–	Desizing of textiles
BAN^TM^	Novozymes	*B. amyloliquifaciens*	Foods and feeds, paper industry
Enzymex (Cocktail),	Exotic Biosolutions Pvt. Ltd.^4^	*B. amyloliquifaciens*	Foods and feeds
Fructamyl® FHT	ERBSLOEH^3^	–	Starch saccharification
Liquozyme® SC DC	Novozymes^6^	Genetically engineered from *B. licheniformis*	Starch saccharification
Natalase®	Novozymes^6^	–	Detergent industry
Stainzyme® plus	Novozymes^6^	Genetically engineered	Detergent industry
Thermamyl®, Takaterm	Novo Nordisk, Denmark^5^	*B. licheniformis*	Detergent industry, paper industry
Validase BAA	DSM Valley Research^2^	*B. amyloliquifaciens*	Food industry
VERON® XTENDER	AB enzymes^1^	–	Baking industry

1 http://www.abenzymes.com

2 www.dsm.com

3 www.erbsloeh.com

4 www.exoticbiosolutions.com

5 www.novonordisk.com

6 www.novozymes.com

7 www.parchem.com

Another application of α-amylases is in textile desizing. During the weaving process the warp (chain) threads are exposed to considerable mechanical strain. In order to prevent breakage, they are usually reinforced by coating (sizing) with a gelatinous substance (size) like starch. Small amounts of fats or oils may be also added to the size, with the aim of lubricating the warp coat surface. As a consequence of the sizing, the warp threads of the fabric are not able to absorb water or finishing agents to a sufficient degree. For that, size must be removed (desizing) before finishing. The complete removal of starch containing size without fiber damage is best obtained by using enzymatic desizing agents. The enzymatic desizing process has three stages. First stage is impregnation. In this stage, enzyme solution is absorbed by the fabric. This stage involves thorough wetting of fabric with enzyme solution at 70°C or higher. An amylase enzyme for desizing must be active at 70°C or higher and optimum pH 5.5–6.5, although efficient desizing have been reported at lower temperatures as well (Cavaco-Paulo and Gübitz, 2003; Chand et al., 2012, 2014). Maximal textile desizing was achieved at 45°C (Chand et al., 2014) and at 60°C (Chand et al., 2012), respectively at pH 4–5. During this stage, gelatinization of the size (starch) is to the highest possible extent. After this, the cloth is incubated under optimum conditions so that the size is broken down by the enzyme. Then, an after wash is performed in which the breakdown products from the size are removed from the fabric. This is best obtained by a subsequent detergent wash (with NaOH) at the highest possible temperature. The enzymes Aquazym 120l, Aquazym Ultra 250l, and Termamyl 60l are available commercially for desizing. The enzymes are commercially available from Novo Nordisk. The advantage of using enzymes in this process is that they are specific for starch, removing it without damage to the support fabric (http://www.novonordisk.com).

Another major application of these enzymes is in baking industry. To prevent the staling of bread and other baked goods, and to improve its texture and shelf-life, the dough is supplemented with various additives (Pritchard, 1992). The bacterial maltogenic α-amylases with intermediate thermostability are known to act as antistaling agents, thereby reducing the crumb firmness during storage (Kumar and Satyanarayana, 2008) by production of malto-oligosaccharides (DP 2–12) and allowing the yeast to act continuously during dough fermentation and early stages of baking. Several studies have proved that supplementation of α-amylases to the dough improves the crumb grain, volume, texture, flavor, and shelf-life of the bread (Van Dam and Hille, 1992; Rao and Satyanarayana, 2007b; Sharma and Satyanarayana, 2010; Amigo et al., 2016). Wild type α-amylase from *G*. *thermoleovorans* is a high maltose forming amylase which improved crumb structure, texture, and shelf life (Rao and Satyanarayana, 2007b). The bread prepared by supplementing the dough with α-amylase of *B. acidicola* had a higher moisture content, reducing sugars and soluble protein than the bread made by using commercial enzyme. It also had high shelf-life of 3 days at room temperature and showed amelioration in the texture and softness (Sharma and Satyanarayana, 2010). Besides these applications, α-amylases are also used for the clarification of haze formed in beer or fruit juices, in the animal feeds for improving the digestibility, in the fields of laundry and dish washing detergents (van der Maarel et al., 2002; Roy et al., 2012; Roy and Mukherjee, 2013).

α-Amylases are also being investigated currently for several other applications such as biodegradation of n-alkanes, synthesis of nanoparticles and others. α-Amylase from *Bacillus subtilis* TB1 was studied for its applicability in the biodegradation of alkanes. The efficiency of biodegradation was better in the presence of starch and the obtained residual hydrocarbons in the system were 53% less than the samples without starch. *In silico* docking of α-amylase with different molecular weight n-alkanes also supported this (Karimi and Biria, 2016). In a study, bioreductive potential of *Micrococcus luteus* for the synthesis of gold nanoparticles (GNPs) was investigated. Extracellular α-amylase and cell wall teichuronic acid (TUA) of *M. luteus* was used in the synthesis of gold nanoparticles. The synthesized GNPs were characterized by UV–VIS spectrometry, transmission electron microscopy (TEM), Fourier transform infrared spectroscopy (FTIR), and dynamic light scattering (DLS; Arunkumar et al., 2013).

## Future perspectives and conclusions

There has been a marked increase in the research and development in the fields pertaining to the enzyme applications in industrial and medical sectors. α-Amylases are being used in several industries for a variety of applications. Cloning, expression, structural studies, and protein engineering of α-amylases from different bacterial and archaeal sources have been carried out for evolving enzymes with the desired characteristics for specific industrial applications. Using newer technologies and approaches, exploration of α-amylases from novel sources must be continued. It is also essential to bring down the cost of enzyme production and meeting consumer demands in all the sectors where this enzyme finds application.

## Author contributions

Both the authors DM and TS have contributed to the data collection and writing this review in its final form.

## Funding

Waiver request has been accepted.

### Conflict of interest statement

The authors declare that the research was conducted in the absence of any commercial or financial relationships that could be construed as a potential conflict of interest.
